# Prevalence and clinical impact of *PIK3CA* and *PTEN* alterations from reflex genomic testing of newly diagnosed colorectal cancer: a population-based series

**DOI:** 10.3389/fonc.2026.1888745

**Published:** 2026-08-07

**Authors:** Laura Kerrin, Osamah Al Haddad, Ellen Reid, John-Joe O’Neill, Lauren McConnell, Anthony Abladey, Paul J. Kelly, Jacqueline A. James, Catherine Davidson, Jack Lee, Catherine Hanna, Maurice B. Loughrey, Mark A. Catherwood, Vicky M. Coyle

**Affiliations:** 1 Northern Ireland Cancer Centre, Belfast Health and Social Care Trust, Belfast, United Kingdom; 2 Regional Molecular Diagnostic Service, Belfast Health and Social Care Trust, Belfast, United Kingdom; 3 Department of Cellular Pathology, Belfast Health and Social Care Trust, Belfast, United Kingdom; 4 The Johnston Centre for Cancer Research, Queen’s University Belfast, Belfast, United Kingdom

**Keywords:** colorectal cancer, non-metastatic, PIK3CA, population series, PTEN

## Abstract

**Background:**

Results from the Adjuvant Low dose Aspirin in Colorectal Cancer (ALASCCA) clinical trial demonstrate a survival benefit associated with aspirin use in patients with non-metastatic colorectal cancer (CRC) harbouring phosphoinositide 3-kinase (PI3K) pathway alterations. Identification of patients in routine clinical care is required for implementation of this therapeutic approach. A real-world analysis of reflex diagnostic testing was performed to assess prevalence of *PI3KCA* and *PTEN* gene alterations and potential clinical impact.

**Methods:**

Next generation sequencing panel testing of all newly diagnosed CRC as part of a Lynch screening service began in the Northern Ireland Cancer Network in 2022. Profiling, using a capture hybridisation assay and custom Small Cancer Panel covers all coding regions of *PTEN* and *PIK3CA* with a limit of detection of 4% variant allele frequency (VAF).

**Results:**

1516 CRC patients underwent genomic profiling at the point of diagnosis from January 2024-March 2025. Across all CRC stages, 170 patients (11.2%) had tumours harbouring *PIK3CA* mutations with the majority occurring in exon 9 and 20. A further 51 of 1516 tumours (3.4%) demonstrated *PTEN* mutations. Of 221 patients with *PIK3CA/PTEN* alterations, 174 (79%) had non-metastatic CRC, of whom 6 (3.4%) had an absolute contraindication to aspirin use. Overall, 168 (11.1%) of 1516 patients in this cohort were therefore identified by this reflex testing pathway as potentially eligible for treatment.

**Conclusions:**

Prevalence of *PIK3CA* and *PTEN* alterations in this series (14.6%) was lower than in the ALASCCA trial (37%) but these results represent a real-world rather than a highly selected trial population. Regardless, our findings support implementing testing for use of a safe, inexpensive repurposed drug and underscore the importance of upfront testing for timely treatment decision-making and implementation of trial findings into routine practice.

## Introduction

Colorectal cancer (CRC) is the third most common cancer and the second leading cause for cancer death worldwide (1). Despite medical advances, recurrence rates after curative intent treatment are substantial with metastatic recurrence in approximately 40% of patients (2).

CRC is characterized by genetic and epigenetic alterations that drive tumour initiation, progression, and metastasis. The phosphatidylinositol 3-kinase (PI3K) pathway is one such pathway that is central to cell survival, proliferation, and growth regulation and is commonly dysregulated in solid tumour malignancies (3). An activating mutation in the *PIK3CA* oncogene is detected in approximately 10-20% of CRC patients; however this varies across tumour stage (4). *PIK3CA* codes for the catalytic subunit, p110⍺, of the PI3K complex, which ultimately activates the serine/threonine protein kinase B (AKT) and mammalian target of rapamycin (mTOR) signalling pathway. Over 80% of *PIK3CA* mutations occur within exon 9 (codons 542/545) which encodes the helical domain, or in exon 20 (codon 1047) which encodes the kinase domain. The prognostic relevance of *PIK3CA* mutations in CRC remains controversial with conflicting studies in the literature in relation to the impact on overall survival (OS) (5, 6).

In addition to *PIK3CA*, the primary role of PTEN (phosphatase and tensin homolog deleted on chromosome ten) is to hydrolyze phosphatidylinositol (3–5)-trisphosphate (PIP3) to phosphatidylinositol (4, 5)- bisphosphate (PIP2), reversing a PIP2 to PIP3 conversion catalysed by PI3K (7). The *PTEN* gene is somatically mutated in tumours but infrequently mutated in cases of CRC with a prevalence of approximately 10% (8).

Acetylsalicylic acid (ASA, aspirin) therapy has been associated with a reduction in CRC development and recurrence (9). However, this finding has not been consistently demonstrated in unselected CRC patients in the adjuvant setting (10, 11). ASA’s mode of action is the inhibition of cyclooxygenase-2 (COX-2) which is overexpressed in CRC. COX-2 facilitates tumorigenesis through the activation of the PI3K signalling pathway (12). Retrospective and observational studies have supported COX2 inhibition as a treatment strategy for inhibiting this signalling in PIK3CA-altered CRC (13).

The recently reported Adjuvant Low dose Aspirin in Colorectal Cancer (ALASCCA) clinical trial has demonstrated that ASA use (160mg daily) in patients with *PI3K* pathway-altered CRC reduced recurrence risk after curative treatment in the stage II/III setting by 50% over a three-year period when compared with placebo (14). Of interest, alterations in PI3K pathways genes (*PIK3CA, PIK3R1* and *PTEN*) were detected in 37% of CRC cases in the ALASCCA cohort. It is well recognized that disparity may exist between the patient cohorts in clinical trials and real-world patient population, related to selection bias (15). Therefore, evaluation in routine clinical care is helpful before implementation of any new therapeutic approach.

In the Northern Ireland Cancer Network (population almost 2 million), all newly diagnosed CRCs are tested for microsatellite Instability (MSI) within the Lynch syndrome screening pathway, using next generation sequencing (NGS) analysis (16, 17). As part of this pathway, *PIK3CA/PTEN* mutational status is available for this population cohort. The aims of this study were to evaluate the genomic landscape of *PIK3CA/PTEN* alterations in our population cohort of CRC to assess the prevalence of alterations and to determine the potential clinical impact for standard care treatment pathways for patients with non-metastatic CRC.

## Methods

### Study cohort and testing pathway

As part of the Northern Ireland Lynch syndrome screening programme, all patients diagnosed with adenocarcinoma of the colon or rectum between January 2024 and March 2025 were eligible for testing. The study cohort covered testing of all patients with CRC within a total population of approximately 1.9 million.

### PIK3CA-PTEN testing

DNA was extracted using the Maxwell^®^ 16 FFPE Plus DNA Kit (Promega, Wisconsin, United States). For NGS, 100ng of extracted DNA was used for library preparation with the KAPA HyperCap Workflow v3.3 (Roche Sequencing solutions, California, United States). DNA libraries were pooled and hybridized with the Precision Medicine Centre of Excellence (PMC, Belfast, UK) bespoke Small Pan Cancer panel and sequenced on the Novaseq 6000 Illumina platform using the NovaSeq SP 50 bp paired-end kit (Illumina, Cambridge, UK) according to the manufacturer’s instructions.

Functional annotation for pathogenic *PIK3CA/PTEN* mutations was collected from the Clinical Knowledgebase (CKB) (https://ckb.jax.org; “loss of function” or “loss of function—predicted”), OncoKB (https://www.oncokb.org; “oncogenic” and “likely oncogenic”) and Clinvar (https://www.ncbi.nlm.nih.gov/clinvar; “pathogenic”or “likely pathogenic”).

Clinical impact assessment:

Consideration of clinical impact was determined by assessment of the proportion of patients meeting the exclusion criteria for the ALASCCA clinical trial and more pragmatically in a real-world population by assessment of the proportion of patients with an absolute contraindication to ASA use based on the British National Formulary (BNF) ASA monograph (https://bnf.nice.org.uk/drugs/aspirin).

### Ethical approval

Using the NHS Health Research Authority decision tool (https://hra-decisiontools.org.uk/research) this study is classed as clinical audit (no randomisation of patients, no alteration to standard clinical care and informs practice in our setting) and therefore formal research ethical approval was not required.

### Statistical analysis

Information related to patient numbers and demographics are presented using descriptive statistics and reported as median and ranges. Characteristics of tumours were compared using the chi-squared test. Statistical analysis was performed using GraphPad Prism (GraphPad software, California, USA).

## Results

### Patient and sample characteristics

Between January 2024 and March 2025, 1516 confirmed CRCs, of all stages, were submitted for MSI testing with subsequent *PIK3CA-PTEN* analysis as part of the reflex NGS pathway. In the study catchment area, approximately 1216 patients are diagnosed with CRC each year, based on Northern Ireland Cancer Registry (NICR) data (18). This equates to approximately 1520 patients expected over the 15-month study period. Therefore, requests for testing in the study period represented 99.7% (1516/1520) of the expected number according to Northern Ireland Cancer Registry statistics, indicating near complete population coverage (18).

### *PIK3CA* and *PTEN* mutation prevalence

170 patients were identified with CRC harbouring PIK3CA alterations (2 patients had synchronous tumours), representing 11.2% (170/1516) of all CRC cases. PTEN mutations were identified in 51 patients (3.4%).

There were no significant differences in clinical characteristics between the *PIK3CA*-mutant CRC and *PTEN*-mutant CRC subgroups. This patient population was representative of the general CRC population as outlined in the Northern Ireland Cancer Registry statistics in terms of age range (median age 70 and 74 respectively) and gender balance (**Table 1**) (18). The majority of patients had stage 1–3 disease (approx. 80%) with distribution of cases between early stage (stage 1-3) and advanced (stage 4) disease consistent with previously reported data from incident CRC diagnoses in the Northern Ireland population (18). *PIK3CA* and *PTEN* mutations were predominantly found in colon cancers, more frequently in right sided rather than left sided cancers, and less frequently in rectal cancers (134 of 172 [77.9%] *PIK3CA-* and 45 of 51 [88.2%] *PTEN*-mutant cancers were colonic). Stage 2 CRC were over-represented in *PIK3CA*-mutant cases (n=60, 35%) relative to previously reported data (NICR as above).

**Table 1:** Clinicopathologic and molecular characteristics of sporadic colorectal cancer according to *PIK3CA* (n=170) or *PTEN* (n =51) mutation status.

Patient Characteristics	*PIK3CA* mutation (n=170 patients) (n=172 tumours)	*PTEN* mutation (n=51 patients)	*P value*
Male (number,%)	83 (49%)	22 (43%)	0.476
Female	87 (51%)	29 (57%)	
Age (years)			0.169
Median	70	74	
Range	34-89	40-91	PIK3CA age 95% CI [66.72,70.21] PTEN age 95% CI [67.83,74.28]
Site			
Right colon	89 (52%)	30 (59%)	
Left colon	45 (26%)	15 (29%)	
Rectum	36 (21%)	6 (12%)	
Unknown	2 (1%)	0	
Stage			
I	31 (18%)	16 (31%)	
II	60 (35%)	16 (31%)	
III	43 (25%)	10 (20%)	
IV	34 (20%)	9 (18%)	
Unknown	4 (2%)	0	
MSI	26 (15%)	15 (29%)	
MSS	145 (84%)	36 (71%)	
Unknown	1 (1%)	0	
No *RAS/RAF* alteration	24 (14%)	14 (27%)	
*KRAS* Mutation	119 (69%)	17 (33%)	
*BRAF* V600E Mutation	21 (12%)	16 (31%)	
*NRAS* Mutation	9 (5%)	2 (4%)	
Unknown	0	0	

### Molecular characteristics

**Figure 1:**
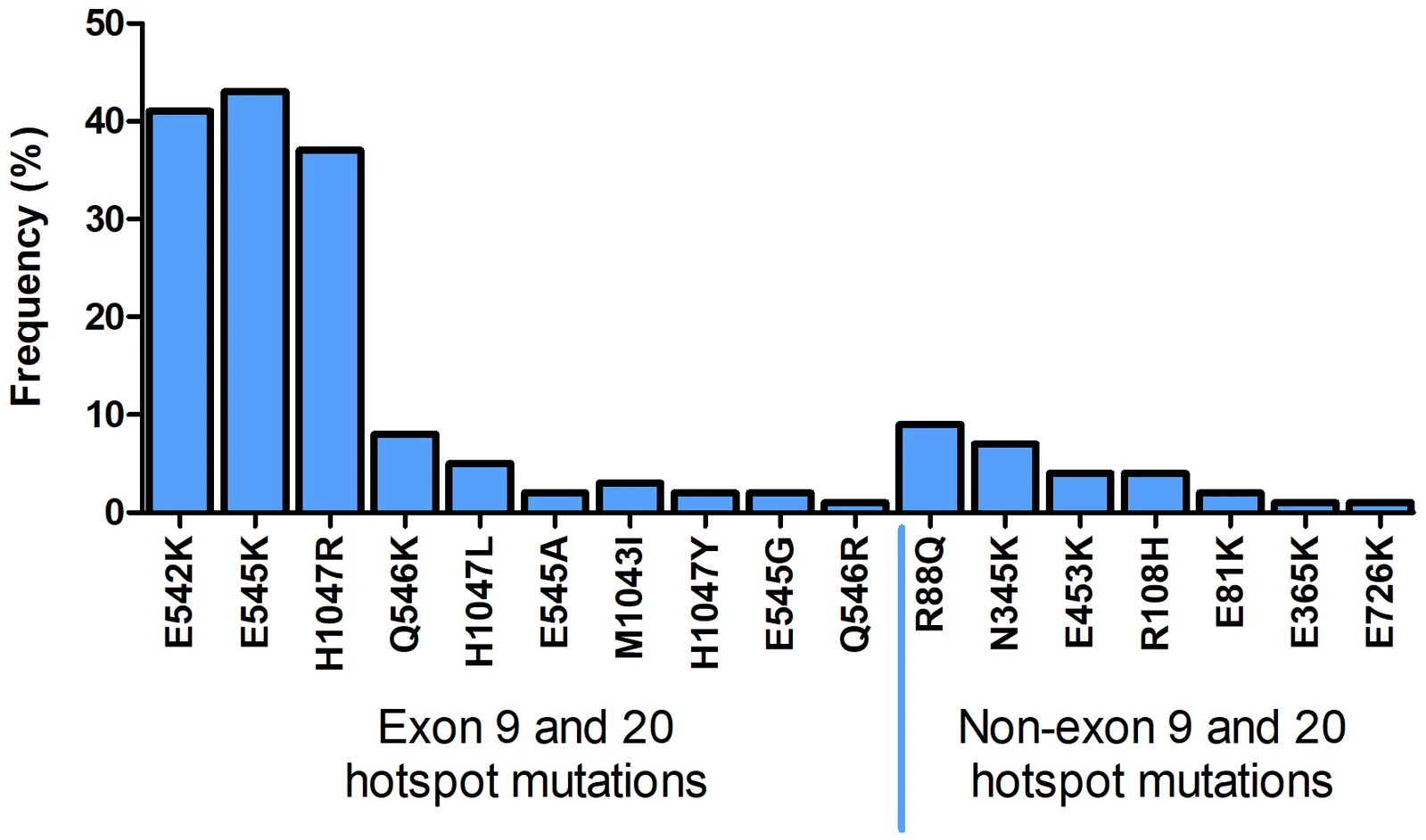
PIK3CA mutation frequency by amino acid.

Of the 172 *PIK3CA* missense mutations identified, 56.4% (97/172) occurred in exon 9 and 27% (47/172) in exon 20. The remaining 28 cases were also classified as pathogenic. (**Figure 1**). Pathogenic mutations at codon 542 (n=41), 545 (n-=47) and 1047 (n=44) which reflect the well-described hotspots in p110alpha accounted for 77% of all exonic mutations.

Among the 51 *PTEN*-mutated tumours, 96% (n=49) had 1 and 4% (n=2) had 2 or more detected somatic mutations (**Table 2**).

**Table 2:** PTEN mutation frequency by amino acid.

Case	Gender	Age	Location	cDNA	Protein
1	M	54	Caecum	c.1008C>A	p.Tyr336*
2	F	45	Caecum	c.607_608delAT, c.517C>T	p.Ile203fs, p.Arg173Cys
3	M	60	Rectum	c.974T>C	p.Leu325Pro
4	M	78	Ascending colon	c.97_99delATT	p.Ile33del
5	F	68	Caecum	c.389G>A	p.Arg130Gln
6	M	69	Rectum	c.493-1G>A	
7	M	61	Sigmoid	c.697C>T	p.Arg233*
8	F	79	Caecum	c.437delT	p.Leu146fs
9	M	81	Caecum	c.801 + 1G>T	–
10	F	71	Ascending colon	c.164 + 1G>T	–
11	F	82	Ascending colon	c.517C>T	p.Arg173Cys
12	F	76	Ascending colon	c.647_648delTG	p.Val216fs
13	M	83	Ascending colon	c.697C>T	p.Arg233*
14	F	56	Sigmoid	c.405delA, c.1027-2A>G	p.Cys136fs
15	F	84	Splenic flexure	c.895G>T	p.Glu299*
16	F	79	Ascending colon	c.800delA	p.Lys267fs
17	M	73	Rectum	c.862G>T	p.Glu288*
18	M	67	Caecum	c.800delA	p.Lys267fs
19	F	80	Caecum	c.188delA	p.Asn63fs
20	F	79	Sigmoid	c.388C>G	p.Arg130Gly
21	M	66	Sigmoid	c.274G>T	p.Asp92Tyr
22	F	82	Caecum	c.389G>A	p.Arg130Gln
23	F	61	Ascending colon	c.313delT	p.Cys105fs
24	M	77	Caecum	c.402_404delGAT	p.Met134del
25	F	76	Sigmoid	c.227_228delAT	p.Tyr76fs
26	M	61	Rectum	c.210-7_210-3delCTTTT	
27	F	91	Caecum	c.284_287delCACC	p.Pro95fs
28	F	45	Sigmoid	c.955_958delACTT	p.Thr319fs
29	M	83	Ascending colon	c.968delA	p.Asn323fs
30	M	60	Sigmoid	c.616_617delTT	p.Phe206fs
31	F	73	Caecum	c.388C>T	p.Arg130*
32	F	67	Ascending colon	c.388C>T	p.Arg130*
33	F	64	Descending colon	c.518G>A	p.Arg173His
34	F	84	Ascending colon	c.968delA	p.Asn323fs
35	M	59	Caecum	c.968delA	p.Asn323fs
36	F	53	Caecum	c.968delA	p.Asn323fs
37	M	70	Sigmoid	c.319G>T	p.Asp107Tyr
38	M	74	Sigmoid	c.176C>A	p.Ser59*
39	M	80	Ascending colon	c.968dupA	p.Asn323fs
40	F	70	Caecum	c.877G>T	p.Gly293*
41	M	84	Ascending colon	c.968delA	p.Asn323fs
42	F	80	Transverse colon	c.333G>A	p.Trp111*
43	M	80	Ascending colon	c.968dupA	p.Asn323fs
44	F	79	Rectum	c.333G>A	p.Trp111*
45	F	71	Descending colon	c.518G>A	p.Arg173His
46	M	40	Sigmoid	c.769T>C	p.Phe257Leu
47	F	75	Hepatic flexure	c.974T>C	p.Leu325Pro
48	F	85	Ascending colon	c.155_157delATG	p.Asp52del
49	F	84	Rectum	c.517C>T	p.Arg173Cys
50	M	40	Hepatic flexure	c.517C>T	p.Arg173Cys
51	F	78	Caecum	c.214G>A	p.Ala72Thr

Microsatellite instability-high (MSI-high) was identified in 41 (19%) CRCs within the overall cohort, in 26 (15%) of *PKI3CA*-mutant and 15 (29%) of *PTEN*-mutant CRCs. Only 2% of patients within this series were known to have Lynch syndrome (LS) at the time of diagnosis. Potential LS cases identified in this study were referred for germline genetic testing in accordance with local guidance. Co-existent *KRAS* mutations were seen in 119 (61%) *PIK3CA*-mutant CRC cases and 17 (33%) *PTEN*-mutant CRC cases. Co-existent *BRAF* V600E mutations were seen in 21 (12%) of *PIK3CA*-mutant cases and 16 (33%) of PTEN-mutant CRC cases.

### Clinical application of testing

Of the 170 patients with *PIK3CA*-mutant CRC, 132 (78%) had non-metastatic CRC (stage 1–3 disease). 22 of these (17%) were already receiving long-term low dose ASA (75mg daily) within its licensed indications for cardiovascular prevention and no contraindication to escalation of ASA dose from 75mg to 160mg daily was identified in any of these patients. 28 patients (21%) would have been deemed ineligible for trial participation, mainly due to long-term treatment with another antiplatelet agent or therapeutic anticoagulant (n=23) and only a minority with recent or active peptic ulcer or gastrointestinal tract bleeding (n=3). Considering concomitant antiplatelet or anticoagulant therapy as a relative contraindication to ASA use in this setting and excluding these patients, 5 of 28 would be considered to have an absolute contraindication due to active peptic ulceration, bleeding, allergy and significant organ dysfunction, representing 3.8% of this cohort in total.

Of the 52 patients with *PTEN*-mutant CRC, 42 (81%) had non-metastatic CRC. 5 (12%) were already receiving low dose ASA, none of which had any contraindication identified for escalation of ASA dose from 75mg to 160mg daily. 14 of 42 (33%) would have been deemed ineligible for trial participation, almost all due to long-term use of another antiplatelet agent or therapeutic anticoagulant. Again, considering concomitant antiplatelet or anticoagulant therapy as a relative contraindication to ASA use in this setting and excluding these patients, 1 of these 42 patients would be considered to have an absolute contraindication to ASA use due to a bleeding disorder.

## Discussion

*PIK3CA/PTEN* gene mutations are well-described in multiple solid tumours, including CRC. Our population-based series of 1, 516 patients with stage 1–4 CRC was screened for somatic mutations in *PIK3CA* and *PTEN*. Within our cohort, we found that the overall prevalence of *PIK3CA* and *PTEN* mutations was 14.6%. *PIK3CA* single-gene alterations were seen in 11.2% of patients with a predominance of mutations in the helical domain (exon 9) over mutations in the kinase domain (exon 20) **Figure 1**. *PTEN* mutations were detected in 3.4% of our cases.

In the existing literature, the prevalence of *PIK3CA* mutations in CRC varies between 3.5-24.7% (4, 19, 20) whilst the prevalence of *PTEN* mutations in CRC has been reported as up to 8.7% (8). Discordant prevalence results likely reflect both ethnic and clinical variables within these studies. For example, it is well documented that *PIK3CA* mutation prevalence in CRC varies between Chinese and European cohorts (21). Also, many of the studies within the literature focus on cohorts with advanced disease CRC rather than a population-based series. The prevalences of *PIK3CA/PTEN* gene mutations in our patient population are lower than those reported in the ALASCCA trial, which suggested around one third of patients with non-metastatic CRC may harbour these alterations. However, our results reflect a prevalence consistent with rates reported in the wider literature and, importantly, a real-world population with newly diagnosed CRC, not a highly selected clinical trial population. An additional contributory factor may be that, in the ALASCCA trial, alterations in the *PIK3R1* gene, involved in the PIK3 pathway, were also examined. *PIK3R1* is not included in the Small Cancer Panel used in the current study (14).

Within our study cohort, *PIK3CA/PTEN-*mutant CRCs were over-represented for colonic, rather than rectal, tumours and for stage 2 disease, compared to the overall cohort data. Co-existent *KRAS* mutations also were more common than expected, compared to the overall cohort. Acknowledging the small sample size within cohort subgroups, others have observed similar trends in relation to colonic location and early stage disease and between concurrent *KRAS* and *PIK3CA* mutations (22, 23) Similarly, patterns of mutational hotspots in *PTEN* associated with MSI-high CRC (8) indicating distinct biology within biologically defined subgroups that may be relevant for novel treatment combinations in the setting of recurrent disease.

The main limitations of the current study are small overall sample size and retrospective collection of clinical data obtained for routine clinical care rather than within the carefully controlled clinical trial environment. Correlation with oncological treatment received and longer-term clinical outcomes is also lacking, preventing any correlation of molecular characteristics of these CRC cases with treatment response or prognosis.

The ALASCCA investigators indicated a potentially significant clinical impact of *PIK3CA/PTEN* mutation testing with a change in treatment pathways through introduction of ASA therapy in up to one third of patients with early-stage CRC. Our data suggest impact for a smaller group of patients, predominantly due to differing prevalence rates with absolute or relative contraindications to ASA use a smaller contributory factor. This again reflects a real-world patient population with multimorbidity rather than a selected clinical trial population. Nonetheless, our findings support clinical impact for a still significant group of patients, approximately 11% of those with early-stage disease with ASA that is safe, inexpensive and given for a time-limited duration. Finally, this study also underscores the importance of upfront testing for timely treatment decision-making and implementation of clinical trial findings into routine clinical use.

## Data Availability

Datasets are available on request: The raw data supporting the conclusions of this article will be made available by the authors, without undue reservation.
